# A structured review of quality of life in advanced and high‐risk cutaneous squamous cell carcinoma shows the need for more studies and better measures

**DOI:** 10.1002/ski2.39

**Published:** 2021-05-07

**Authors:** R. Starkings, V. Shilling, V. Jenkins, L. Fallowfield

**Affiliations:** ^1^ Sussex Health Outcomes Research and Education in Cancer (SHORE‐C) Brighton and Sussex Medical School University of Sussex Brighton UK

## Abstract

**Background:**

Cutaneous squamous cell carcinoma (cSCC) accounts for nearly a quarter of non‐melanoma skin cancers. Studies reporting Quality of Life (QoL) in this group focus on early stage disease. A small proportion of cSCC patients have high‐risk or advanced disease, with potentially significant QoL impacts, yet are largely overlooked.

**Aims:**

This structured review appraises measures and published QoL outcomes in this group.

**Materials & Methods:**

We conducted searches in MEDLINE, EMBASE, CINAHLplus and PsycInfo in June 2020 (updated in October) to identify publications specifically reporting QoL outcomes in this cohort. Returns were reviewed against a strict set of eligibility criteria.

**Results:**

We identified seven publications for inclusion; three relating to high‐risk cSCC, three to metastatic disease and one to unresectable disease. Publications were appraised for quality using the Mixed Methods Appraisal Tool. Only one fulfilled more than two of the five quality criteria. Studies employed a range of patient reported outcome measures to assess QoL, both generic and disease specific.

**Discussion:**

All studies with multiple time‐points reported stable or improving QoL, however extrapolation of these findings to the cSCC population is not warranted due to study limitations including mixed populations, incomplete data sets or single measurements. We set out to review the QoL literature for high‐risk and advanced cSCC and found a small and disparate body of evidence. Studies varied significantly in terms of study population, design and quality. While the identified studies suggested stable or improving QoL, we question the choice of measures used and highlight the need for further work in this area.

**Conclusion:**

While there are some published reports about quality of life for patients with early stage cutaneous squamous cell carcinoma, these impacts for the high‐risk or advanced cohort are largely unexplored. We conducted a structured review of published measures and outcomes used in this cohort and found a demonstrable need for further, targeted, exploration of patient needs in this area.

## Graphical abstract

While there are some published reports about quality of life (QoL) for patients with early stage cutaneous squamous cell carcinoma (cSCC), these impacts for the high‐risk or advanced cohort are largely unexplored. We conducted a structured review of published measures and outcomes used in this cohort and found a demonstrable need for further, targeted, exploration of patient needs in this area.

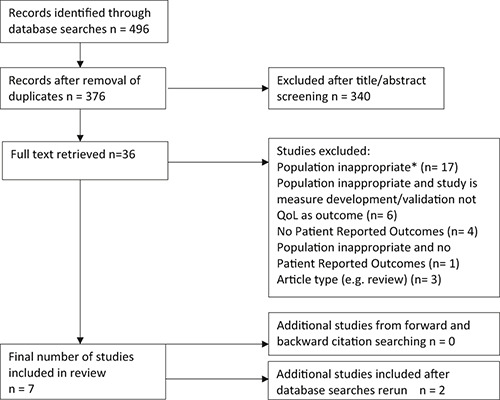

1

**What is already known about this topic?**

- There is a small cohort of patients with cutaneous squamous cell carcinoma who will be classified as advanced or high‐risk.
- While there are some published reports about quality of life (QoL) for patients with early stage disease, these impacts for the high‐risk or advanced cohort are largely unexplored.

**What does this study add?**

- After reviewing the published measures and outcomes used in this cohort, there is demonstrable need for further, targeted, exploration of patient needs in this area. This can then inform the creation of well‐validated outcome measures.

## 2 INTRODUCTION

Over 100 000 cases of non‐melanoma skin cancer (NMSC) are diagnosed each year in the UK. Cutaneous squamous cell carcinoma (cSCC) is the most common after basal cell carcinoma (BCC), accounting for around 23% of NMSCs, more common in men and the elderly and often found on the head and neck.^1^, ^2^ In a majority of cases (∼95%) surgical treatment alone is curative but around 5% will require more complex treatments.^3^ A small percentage of patients develop metastatic or locally advanced disease and have a poor prognosis with a 10‐year survival rate <20% with regional lymph node involvement and <10% in the presence of distant metastases.^4^ A subset of cSCC patients are classified as high‐risk, with disease that is poorly controlled with conservative treatment and a greater propensity for metastasis.^5^ Features of the tumour such as size, site, speed of growth, and depth of invasion or differentiation, alongside factors like immunosuppression, may contribute to this. Identifying high‐risk cSCC patients is in itself complex with definitions and poor stratification from previous staging systems.^6^, ^7^

Quality of Life (QoL) for patients with NMSC is reduced due to disease symptoms, treatment side‐effects, its impact on daily living,^8^ future cancer worries, concerns about appearance,^9^, ^10^, ^11^, ^12^ skin cancer specific and general distress,^11^, ^13^, ^14^, ^15^ and unmet supportive care needs.^13^ With the most common site of disease being head and neck, the cosmetic outcomes can have substantial psychosocial comorbidity. This can stem from the tumour but also from treatment, impacting confidence, distress and body image.^13^ Body image and social support are reported to play a mediating role in QoL.^16^ Some studies note an association with age and gender, particularly around appearance,^17^ although these are not consistent predictors of QoL.^8^ Studies also suggest QoL improves over time in non‐metastatic cancers,^15^, ^18^, ^19^, ^20^ with pre‐treatment QoL strongly predicting post‐treatment QoL.^21^ Many concerns identified in quantitative studies, such as appearance, physical and social impacts, satisfaction with care, and new or recurrent cancers have been echoed in qualitative research.^22^

There are challenges when interpreting this literature due to methodological differences. Most QoL studies in NMSC combine BCC and cSCC. However, the majority of patients have BCC, with different treatments and outcomes to cSCC. Where results are not presented separately for patient groups, interpretation of findings is difficult, particularly within small samples. The majority of studies have not included advanced/high‐risk cSCC or have omitted disease characteristics of the population.

Overall, publications show large ranges in the type and magnitude of QoL effects associated with diagnosis and treatment for non‐metastatic NMSC. Some of this variability stems from the measures used. Vinding^23^ suggested that studies employing generic and dermatology specific measures demonstrate minimal impact of NMSC on QoL (e.g., Arts et al.,^24^) yet qualitative studies,^22^ and those using open‐ended questions,^12^ have identified various issues, particularly emotional concerns. This underlines the need for careful selection of outcome measures with sensitivity to the multi‐faceted influences faced by patients.

Whilst informative, the direct relevance to the advanced/high‐risk cSCC population of QoL studies conducted with early stage or mixed samples is questionable as disease and treatment characteristics are qualitatively different for patients receiving curative surgery, for example survival concerns. Without measures specifically designed for the advanced/high‐risk cohort, it remains unclear whether existing tools provide sufficient coverage and granularity for them.

We did not identify any publications specifically reviewing studies reporting QoL in patients with advanced/high‐risk cSCC. To address this, our structured review addressed two broad research questions:

1. What reports are there examining the QoL of patients with high‐risk, locally advanced, or metastatic cSCC?
2. What outcome measures have been used to measure QoL?

## 3 METHODS

### 3.1 Search strategy

Groups of free text search terms were generated based on condition, stage, and quality of life. Terms within each group were combined with Boolean ‘or’ string, groups were then combined with ‘and’. See Supporting Information for the search strategy as run in MEDLINE, adapted for other databases. Searches were run in MEDLINE, EMBASE, CINAHLplus and PsycInfo on 18 June 2020. Searches were not limited by design or date but were restricted to articles in the English language.

### 3.2 Study selection criteria

Articles were assessed against eligibility criteria (Table 1):

1. Population: must be, at least in part, high‐risk, locally advanced, or metastatic cSCC. As previously noted, the classification of ‘high‐risk’ is variable. Therefore, we included papers that self‐defined their population as such.
2. Intervention: no or any intervention is acceptable.
3. Comparator: no or any comparator group is acceptable.
4. Outcome: must report QoL either through Patient Reported Outcomes or in a qualitative study.

**TABLE 1:** Inclusion and exclusion criteria

Inclusion	Exclusion
Patients with high‐risk, locally advanced or metastatic cutaneous squamous cell carcinoma	Papers where population has other type of cancer or other type of SCC or where cancer is curable with surgery and or radiotherapy
Papers with adult populations (>18)	Paediatric population
Qualitative or quantitative papers reporting on QoL or patient experience	Papers reporting adverse events/side effects only i.e. no patient reported outcomes Papers where clinician reporting rather than patient reporting is recorded Studies which are solely measure development/validation rather than QoL as an Outcome Article is a review paper, case report or book chapter

Abbreviation: Qol, quality of life.

Any study type was eligible for inclusion. Conference abstracts were included if sufficient data were provided, as were research letters. Book chapters, review papers and case reports were excluded.

**FIGURE 1:**
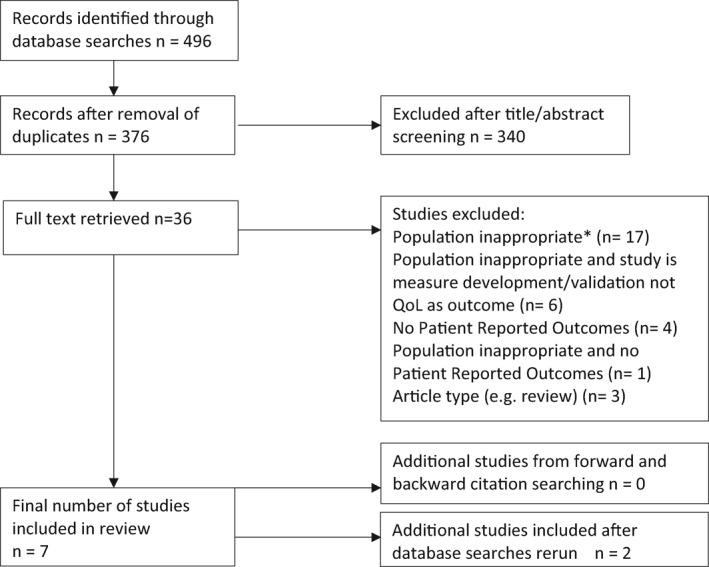
PRISMA‐style diagram. *Population is not, or does not include high risk/advanced/metastatic cSCC or the disease characteristics of population not properly defined or groups not differentiated in results reporting

Backwards citation chasing (one generation) using references of the included studies and forwards citation chasing (one generation) via Web of Science identified no additional eligible studies. Searches were rerun on 20 October 2020 in case of new publications, of which two were identified. See Figure 1 for a PRISMA style flow‐chart of study selection.

### 3.3 Quality appraisal of included studies

Studies were assessed for quality using the Mixed Methods Appraisal Tool (MMAT).^25^, ^26^, ^27^ The MMAT was developed specifically as a critical appraisal tool for systematic reviews including differing study designs. The MMAT comprises two screening questions and five items appraising different study categories. Studies were independently assessed by two authors (VS/RS). Disagreements were resolved through discussion. Studies were not excluded on the basis of quality however we were mindful of study limitations and how this might affect our interpretation.

### 3.4 Data extraction and synthesis

For each included paper, the following data were extracted and tabulated: study type, sample characteristics, PRO measures used (where appropriate), outcomes measured, and outcomes reported. Data was extracted by one author (VS) and checked by a second (RS). Our a priori assumption was of a small and disparate body of literature and as such, meta‐analysis was not planned. Findings were instead brought together with descriptive synthesis.

## 4 RESULTS

Searches identified 445 records. Combining and de‐duplicating these resulted in 357. After screening title and abstract, 36 full records were considered against eligibility criteria. Five studies were originally included in the review and a further two added when database searches were rerun (Figure 1). They comprised: three conference abstracts,^28^, ^29^, ^30^ one conference presentation published as a full manuscript,^31^ one research letter^32^ and two primary research articles.^33^, ^34^ Three publications pertain to high‐risk cSCC,^29^, ^32^, ^33^ three to metastatic cSCC,^28^, ^30^, ^31^ and one to unresectable disease including locally advanced patients and those with distant metastases.^34^ Two of the publications, a conference abstract^29^ and primary research paper^33^ report the same feasibility data and will be discussed as one, though due to slight variations in the publications, they are presented separately in Table 2, which describes study characteristics.

**TABLE 2:** Study characteristics

Author/date	Publication type	Study design	Sample Size	Sample characteristics	QoL outcome measures	QoL outcomes reported	Key finding
Hughes et al. 2020^27^	Conference abstract	QoL data from clinical trial of pembrolizumab. QoL data collected at baseline, wk3 and wk6 then every 6 weeks for yr1, every 9 weeks yr2, and 30 days safety visit. Primary analysis presented here was at week 12.	*n* = 105	Patients with recurrent or metastatic cSCC. Median age 72.	EORTC QLQ‐C30^30^ EQ‐5D^29^	Primary analysis was mean change from baseline at week 12; improvement was defined as ≥ 10 point increase from baseline. Data reported for 99 patients for EORTC QLQ‐C30 and 100 patients for EQ‐5D. Week 12 change from baseline was stable for global health status (GHS)/QoL (4.95 points; 95% CI 1.00–10.90). For physical function (−3.38, 95% CI 8.80– 2.04) For EQ‐5D VAS (1.97, 95% CI 3.85–7.79). This trend was sustained through week 48. Using the 10 point criteria, 29.3% (95% CI 20.6 ‐ 39.3) of patients improved on GHS/QoL and 17.2% (95% CI 10.3–26.1) for physical function.	Pembrolizumab showed a clinically meaningful objective response rate without meaningful impact on overall HRQoL.
Maubec et al. 2020^33^	Primary research	QoL data from clinical trial of pembrolizumab. QoL data is reported at baseline and week 15.	Primary cohort *n* = 39 Expansion cohort *n* = 18 Local or regional disease *n* = 43 Distant metastases *n* = 14	Patients with unresectable cSCC. Median age 79 years (range 42–99)	FACT‐G^31^	Authors report a non‐significant improvement in FACT‐G scores between baseline and week 15 (74.6 ± 14.1 to 79.5 ± 14.0, *p* = 0.11). Analysis compared FACT‐G change reported by participants who responded to treatment with those who did not respond. Authors report meaningful improvement in the responder group compared to the non‐responder group mean difference 6.5 ± 9.9 versus. 1.6 ± 16.8, *p* = 0.025). 56/57 patients completed QoL at baseline. 36/45 evaluated at week 15 completed QoL. The number of responders and non‐responders in the group of 36 patients who completed QoL at week 15 is not reported.	QoL was not a primary outcome of this manuscript. Overall, QoL showed a non‐significant improvement between baseline and 15 weeks of treatment with pembrolizumab. Mean difference scores were significantly larger in the responders group than the non‐responders.
Migden et al. 2020^29^	Conference abstract	QoL data from clinical trial of cemiplimab. QoL data collected at baseline and day 1 of treatment cycles 1 to 5.	*n* = 193 Metastatic group *n* = 115 Locally advanced group *n* = 78	Patients with locally advanced or metastatic cSCC, ≥1 lesion, ECOG performance ≤1	EORTC QLQ‐C30^30^	QLQ‐C30 completed at baseline and day 1 of cycles (C) 1 through 5. Primary outcome was mean change, estimated by MMRM modelling, from baseline to C5. Clinically meaningful change was prespecified as ≥10 points. Clinically meaningful improvement was seen for pain score (least squares mean [standard error] change −12.1 [2.1]; *p* < 0.0001). Other domains/items analysed were stable or showed non‐significant trend towards improvement. 85%–94% of patients remained stable or reported clinically meaningful improvement on individual symptoms: dyspnoea, nausea/vomiting, diarrhoea, constipation, appetite loss. N.B. results are combined for metastatic and locally advanced patients and only 99/193 patients appear to have QoL data at baseline and C5.	Most patients treated with cemiplimab improved or maintained HRQOL; pain specifically showed clinically meaningful reduction.
Wali et al. 2020^32^	Primary research	Feasibility study. QoL data collected at baseline and 3 months for all participants and at 6–9 months for high‐risk participants.	*n* = 318 consented, *n* = 279 complete baseline: 196 in the low‐risk group, 83 in high‐risk.	Patients with histologically demonstrated low‐risk NMSC (*n* = 196, mean age 72 years; 114 male/82 female; 171 [87.2%] BCC/20 [10.2%] SCC/5 [2.5%] other) Or high‐risk NMSC (*n* = 83; mean age 78 years; 57 male/26 female; 100% SCC)	Skin Cancer Quality of life impact tool (SCQOLIT)^33^ EQ‐5D^29^ A sub‐set of participants and some clinical staff were also invited to take part in a semi‐structured interview	Response rates at baseline were (group 1, 90.3%, *n* = 196; group 2, 82.1%, *n* = 83) and declined at 3 months (group 1, 80.6%, *n* = 171; group 2, 69.3%, *n* = 70) and 6–9 months (group 2, 58.4%, *n* = 59). Missing data at baseline was <0.3%. Total scores were only calculated for those with complete data (*n* = 274) Total scores showed no ceiling effects (poorest QoL); floor effects were present, 13.9% (*n* = 38) reported score of zero (highest possible QoL) Seven (2.6%) reported scores above the threshold set for clinically significant QoL impairment at baseline (4 [2.1%] in the low‐risk group, 3 [3.7%] in the high‐risk), four at 3 months (3 [1.7%] in low‐risk, 1 [1.5%] in high‐risk and one at 6–9 months (1 [1.7%] in high‐risk group, low‐risk did not complete this time point). Groups did not differ on SCQOLIT scores at baseline Baseline to 3 months SCQOLIT scores (both groups combined for those with a complete dataset) showed significant improvement (*n* = 235, baseline mean 5.05 [SD 5.16], 3 months mean 3.69 [SD 4.36], t = 4.49, *p* < 0.001). SCQOLIT showed good internal consistency Cronbach's alpha = 0.84 (*n* = 274).	Total SCQOLIT scores were low (high QoL) for both groups and significant improvement was seen over a 3‐month period. A small proportion showed clinically significant QoL impairment. Analysis was conducted for combined groups; not possible to comment on high‐risk patients specifically.
						Mean EQ‐5D score was 0.88, SD 0.18, *n* = 259. Correlation with SCQOLIT total scores at baseline was weak for dimensions considered unrelated to SCQOLIT content (mobility *r* = 0.13, *p* = 0.032; self‐care *r* = 0.07, *p* = 0.287) and weak‐moderate for those dimensions most similar (anxiety/depression *r* = 0.42, *p* < 0.001; usual activities *r* = 0.20, *p* = 0.001; pain/discomfort *r* = 0.28, *p* < 0.001). Overall correlation with EQ‐5D *r* = −0.30, *p* < 0.001. Interview data pertained to acceptability and feasibility of using the SCQOLIT measure and does not contribute QoL data so is excluded.	
Wali et al. 2017^28^	Conference abstract	Feasibility study. QoL data collected at baseline and three months for all participants and at 6 months for high‐risk participants.	Low‐risk *n* = 196 High‐risk *n* = 82	Patients with histologically demonstrated low‐risk NMSC (mean age 72.5 years; 114 male/82 female; 87.2% BCC/10.2% SCC) Or high‐risk NMSC (mean age 77.8 years; 57 male/25 female; cancer type not stated)	Skin Cancer Quality of life impact tool (SCQOLIT)^33^ EQ‐5D^29^	Groups did not differ on SCQOLIT scores at baseline Baseline to 3 months SCQOLIT scores (both groups combined) showed significant improvement (*n* = 231, mean improvement 1.31, t = 4.46, *p* < 0.001). SCQOLIT showed good internal consistency Cronbach's alpha = 0.84 (*N* = 273) and convergent validity against the EQ‐5D (*p* < 0.001).	Total SCQOLIT scores were low (high QoL) for both groups and significant improvement was seen over a 3‐month period.
Wang et al. 2013^30^	Conference presentation of primary research	Cross sectional survey, consecutive patients approached. QoL data collected at one time point only.	*n* = 42	Patients at least 6 months after treatment for metastatic cSCC of head and neck; 35 male/7 female; mean age 71 (range 50–88); surgery alone *n* = 3, surgery + radiotherapy *n* = 27, surgery + radiochemotherapy *n* = 11, radiotherapy alone *n* = 1	Functional assessment of Cancer therapy – Head and neck (FACT‐H&N)^31^ ^,^ ^32^ Facial disability index (FDI)^36^	FACT‐H&N Mean total general score: 91 ± 13 (range 44–108) Mean total H&N subscale: 32 ± 5 (range 20–40) Mean overall total score: 124 ± 17 (range 64–148) FDI Mean physical function: 89 ± 15% (range 45%–100%) Mean social function and wellbeing: 76 %± 12% (range 52%–100%) Women reported significantly poorer FDI physical function (90 ± 22% vs. 100 ± 12%, *p* = 0.017) Patients who had consumed alcohol in the preceding 7 days (*n* = 24 [57%]) reported significantly higher scores on FACT social (26 ± 3 vs. 23 ± 5, *p* = 0.016); general (93 ± 10 vs. 90 ± 15, *p* = 0.041) and total scores (128 ± 13 vs. 120 ± 20, *p* = 0.033) and FDI physical (100 ± 11% vs. 88 ± 17%, *p* = 0.034) Most commonly reported symptoms: dry mouth (32 patients, 76%); change in voice (23 patients, 55%); unhappy with appearance of face and neck (19 patients, 45%); unable to eat food they liked (17 patients, 40%); pain in mouth, throat or neck (17 patients, 40%) Chemotherapy was not associated with worse QoL–note treatment completed at least 6 months prior	The authors note that females reported worse physical function QoL while participants who drank alcohol reported better QoL than those who did not. It is hard to draw meaningful conclusions due to limitations of study design.
Yan et al. 2019^31^	Research letter	Prospective study – primary outcomes were local recurrence and QoL after surgery and adjuvant radiotherapy. QoL data collected before and after radiotherapy.	*n* = 52	Patients with high‐ risk aSCC (defined by depth of invasion > 6 mm or desmoplasia)	Skindex‐16^34^ ^,^ ^35^	Skindex‐16 score available from 26 (50%) of patients before adjuvant radiotherapy and 24 (46%) after. Composite score improved by 11.2 points (95% CI 2.0–20,4, *p* = 0.02). Symptoms domain: 3.9 (95% CI 7.8–15.5, *p* = 0.51); Emotions domain: 15.0 (2.3–27.8, *p* = 0.02); Functioning domain: 11.7 (2.1–21.3, *p* = 0.02)	Surgery and adjuvant radiotherapy was associated with low risk of local recurrence with no deleterious effect on QoL.

Abbreviations: cSCC, Cutaneous squamous cell carcinoma; ECOG, Eastern Cooperative Oncology Group; EORTC‐QOL, European Organization for the Research and Treatment of Cancer Quality of Life Questionnaire; EQ‐5D, EuroQol‐ 5 Dimension; FACT‐G, Functional Assessment of Cancer Therapy‐General; NMSC, non‐melanoma skin cancer; QLQ, quality of life questionnaire; Qol, quality of life; SCQLIT, Skin Cancer Quality of Life Impact Tool.

### 4.1 Types of study and quality appraisal

Four studies reported QoL data before and after treatment. Three presented QoL data from a clinical trial, two of pembrolizumab,^28^, ^34^ one cemiplimab.^30^ In all cases, QoL data was collected across multiple time‐points and all participants received the investigational product. In two publications, participants were treated as a single group for analyses.^28^, ^30^ The third categorized participants as treatment responders and non‐responders, for QoL analysis.^34^ The other study^32^ reported QoL before and after standard of care post‐surgery radiotherapy.

The linked article and conference abstract by Wali and colleagues^29^, ^33^ report a feasibility study measuring QoL at distinct time‐points not associated with treatment intervention, allowing for some comparison of patient groups (low vs. high‐risk), although most analyses were conducted across groups.

Finally, the descriptive quantitative study published by Wang^31^ collected QoL data from participants at a single time‐point, at least 6 months post‐treatment.

Most of the studies included in the review were appraised using the Quantitative Non‐Randomized category of the MMAT. The aforementioned study by Wang^31^ was appraised using the Quantitative Descriptive category. Table 3 provides the ratings for each study. One of the Wali publications^33^ included qualitative interviews, however these pertained only to the acceptability and feasibility of the SCQOLIT measure so were excluded. The remaining QoL measurement was evaluated with the Quantitative Non‐Randomized category of the NMAT.

**TABLE 3:** Individual item ratings for each study using the MMAT^a^

Study	Methodological quality criteria																				
	Are there clear research questions?			Do the collected data allow to address the research questions?			Are the participants representative of the target population?			Are measurements appropriate regarding both the outcome and intervention (or exposure)?			Are there complete outcome data?			Are the confounders accounted for in the design and analysis?			During the Study period, is the intervention administered (or exposure occurred) as intended?		
	Yes	No	Can't tell	Yes	No	Can't tell	Yes	No	Can't tell	Yes	No	Can't tell	Yes	No	Can't tell	Yes	No	Can't tell	Yes	No	Can't tell
Hughes et al. 2020^27^	✓			✓					✓	✓			✓					✓			✓
Maubec et al. 2020^33^	✓			✓			✓			✓				✓				✓	✓		
Migden et al. 2020^29^	✓			✓					✓	✓				✓				✓			✓
Wali et al. 2020^32^ ^,^ ^b^/Wali et al. 2017^28^	✓			✓			✓			✓				✓			✓				✓
Yan et al. 2019^31^	✓			✓					✓	✓				✓				✓			✓

	Are there clear research questions?			Do the collected data allow to address the research questions?			Is the sampling strategy relevant to address the research question?			Is the sample representative of the target population?			Are the measurements appropriate?			Is the risk of nonresponse bias low?			Is the statistical analysis appropriate to answer the research question?		
	Yes	No	Can't tell	Yes	No	Can't tell	Yes	No	Can't tell	Yes	No	Can't tell	Yes	No	Can't tell	Yes	No	Can't tell	Yes	No	Can't tell
Wang et al. 2013^30^	✓				✓			✓				✓	✓					✓		✓	

Abbreviation: MMAT, Mixed Methods Appraisal Tool.

^a^ Appraised independently by two reviewers (VS/RS), discrepancies resolved through discussion.

^b^ Note, one of the Wali publications^27^ included qualitative interviews as well as QoL measurement, and so would be considered a mixed methods study, however interview data pertained only to acceptability and feasibility of using the SCQOLIT measure and does not contribute QoL data so was excluded. The QoL measurement was evaluated with the Quantitative Non‐Randomized category of the MMAT.

All studies were deemed to have clear research questions, aside from the Wang study^31^ which failed the second screening question of whether data collected allowed the research questions to be addressed. However, we were able to appraise the study against the remaining criteria. While the majority of studies used appropriate measures, there was a concern that in a number of cases there were incomplete data, where participants were lost to follow up. In relation to confounders, intervention fidelity and the representativeness of the study population, it was simply not possible to rate most studies (Table 3). This was due to participants lost to follow up. We also acknowledge that, in the case of two studies in particular,^28^, ^30^ this reflects that the publications were conference abstracts and information available was inevitably limited.

### 4.2 Measures used

Studies employed a number of QoL measures, some generic such as the EQ‐5D,^28^, ^29^, ^33^ some designed to measure HRQoL in cancer generally, such as the EORTC QLQ‐C30^28^, ^30^ and FACT‐G^34^ or more specifically, such as the FACT‐H&N.^31^

The EQ‐5D is a widely used measure of generic health related QoL, comprising 5 dimensions: mobility, self‐care, usual activities, pain/discomfort and anxiety/depression.^35^ It provides a utility score for health economics with a cursory QoL assessment. The EORTC QLQ‐C30 contains 30 items measuring QoL in cancer patients.^36^ It has five functional scales (physical, role, cognitive, emotional, and social functioning), a global QoL scale, three symptom scales (fatigue, nausea and vomiting, and pain), and six single items (appetite loss, diarrhoea, dyspnoea, constipation, insomnia, financial impact). The FACT general scale contains 27 QoL items in four domains: physical well‐being, social and family well‐being, emotional well‐being and functional well‐being.^37^ The Head and Neck subscale contains 11 further items relevant to head and neck cancer symptoms.^38^

Several studies used measures capturing QoL issues related to dermatology or skin cancer, namely the SCQOLIT,^29^, ^33^ Skindex‐16^32^ and the Facial Disability Index (FDI).^31^ The Skin Cancer Quality of Life Impact Tool (SCQOLIT)^39^ was developed and evaluated in melanoma and NMSC patients. It is a single scale with 10‐items relating to recurrence, appearance, social and emotional impacts, communication with HCPs and sun behaviour. The Skindex‐16, has 16 items in three scales: emotions, functioning and symptoms.^40^, ^41^ It was not designed specifically for skin cancer. The same is true of the FDI, a 10‐item scale of facial motor disorder^42^ with two domains; physical function of the facial nerve and social function and wellbeing.

In summary, the measures used in these studies were not developed and/or validated specifically for the high‐risk or advanced cSCC patient groups.

### 4.3 Quality of life outcomes reported

Yan and colleagues^32^ report QoL (using Skindex‐16) for high‐risk patients receiving radiotherapy after surgery. Their study obtained QoL data from 26 patients prior to radiotherapy and 24 after. Consistent with other studies, QoL scores improved after treatment. Total scores significantly improved as did two of the three domains (emotions and functioning).

Hughes^28^ reports QoL data from 100 patients with recurrent or metastatic cSCC receiving pembrolizumab as part of the KEYNOTE‐629 trial. QoL was measured using the EORTC QLQ‐C30 and EQ‐5D‐5L. At week 12 mean change from baseline was small and the authors conclude that overall Global Health Status/QoL and physical function were stable; a trend continuing to 48 weeks. A proportion of patients reported improved scores; 29.3% for GHS/QoL and 17.2% for physical functioning. The authors conclude that pembrolizumab has clinically meaningful benefit for this group without impacting overall QoL. Generic measures may lack specificity for issues faced by these patients, potentially explaining stable scores. The QoL data has yet to be fully published in a peer reviewed journal.^43^ While efficacy data from KEYNOTE‐629 has been published,^43^ the QoL data has yet to be made available in full in a peer reviewed journal. In addition, there are concerns as to the sensitivity of generic HRQoL and health utility measures to the specific QoL issues faced by this patient group which potentially explains stable QoL scores.

Maubec^34^ reports a phase 2 study of first‐line pembrolizumab for patients with unresectable cSCC. The FACT‐G was used at baseline and after 15 weeks of treatment. Fifty‐six participants completed baseline assessment, only 36 had data at week 15. The authors report a non‐significant improvement in QoL between these time‐points. Subgroup analysis comparing FACT‐G change for treatment responders and non‐responders shows that mean difference scores were significantly larger in the responders than the non‐responders. Unfortunately, QoL was a secondary objective in a publication reporting the main trial findings of response rate and survival. Very little attention is given to QoL and the analysis reported is cursory.

Migden and colleagues^30^ report QoL data from a phase 2 clinical trial of cemiplimab in metastatic or locally advanced cSCC. This was measured using the EORTC QLQ‐C30 and EQ‐5D. One hundred and ninety‐three patients participated, however only 99 had QoL data at follow up. Patients reported low symptom burden at baseline and the majority of scores on key QoL domains remained stable or showed clinically meaningful improvement.

Wang and colleagues^31^ report a small, cross sectional study of 42 patients treated for metastatic cSCC of the head and neck. Patients were considered disease free following different combinations of treatment completed at least 6 months prior. As a cross‐sectional study, the QoL scores, measured via the FACT‐H&N and FDI, have limited value, though it is noteworthy that FACT‐G scores were somewhat higher than reported population norms. The authors report no association between a number of variables and QoL including: marital status, education, employment, prior chemotherapy, and time since treatment. In line with common concerns following treatment for head and neck disease, dry mouth was reported by a majority of patient (32 patients, 76%), as was a change in voice (23 patients, 55%). A large proportion reported that they were unhappy with the appearance of face and neck (19 patients, 45%), were unable to eat the food they liked (17 patients, 40%) or had pain in the mouth, throat or neck (17 patients, 40%).

In the linked non‐interventional publications by Wali and colleagues,^29^, ^33^ participants with NMSC were categorized as high or low risk. Quality of life was assessed using the SCQOLIT and EQ‐5D at baseline and three months. High‐risk participants also completed measures after 6–9 months. Participant groups did not differ on baseline QoL, thereafter QoL data was combined across groups. Quality of life improved significantly between baseline and 3 months. Overall, both groups had low total SCQOLIT scores, suggesting limited QoL impacts. A small proportion (2.6%) did however report high burden, scoring above a threshold determined by the measure developers. This was not specific to the high‐risk group.

## 5 DISCUSSION

There are few publications examining the QoL of patients with high‐risk and advanced cSCC. This structured review identified merely seven relevant publications. Viewed together, QoL appeared stable or to improve over time, however meaningful comparisons across studies were impossible due to the heterogeneity of factors including patient samples, QoL measures used and different treatment interventions. Notably, the measures used were not developed and validated specifically in these patient groups and, as three of the publications relate to conference presentations and one a research letter, the full QoL data are not yet published for comparison. Furthermore, only one study was rated positively on more than two of the five quality criteria while four lacked complete outcome data. Proportionally, this is a small cohort of patients comprising mainly older men, already historically underrepresented and neglected in QoL studies.^44^

The recent approval of novel therapies may raise the profile of this group. Indeed, three of the publications in this review pertain to trial data for cemiplimab^30^ and pembrolizumab,^28^, ^34^ with real world studies planned to include QoL assessment.^45^ Few RCTs have been carried out in this group; previous ambiguity around classification of risk may have contributed to this. The British Association of Dermatologists (BAD) has recently issued new guidance as to how to classify patients as ‘high’ or ‘very high’ risk.^7^ This should allow further definition of this cohort within research.

### 5.1 Measuring QoL in advanced and high‐risk cSCC

There are limited, validated, QoL measures designed for patients with NMSC in general,^46^ and none for the advanced or high‐risk cSCC group.

The Skin Cancer Index (SCI) has been identified as a potentially useful measure for patients with cSCC in a number of systematic reviews.^46^, ^47^, ^48^, ^49^ This is a well‐validated 15‐item disease specific instrument with three subscales: emotion, social, and appearance.^9^, ^10^ The SCI was not used by any study included in our review. Other validated scales worth considering include the Dermatology Life Quality Index (DLQI), with 10‐items focusing on daily activities and relationships.^50^, ^51^ However, it was not developed specifically for skin cancer and may not capture all relevant issues. The FACE‐Q Skin Cancer Module,^52^ includes items regarding cancer worry, but its emphasis on appearance following surgery for early cancer makes it unlikely to be useful with advanced patients. This is true of the Patient Outcomes of Surgery‐Head/Neck.^53^

The SCQOLIT^39^ has recently been developed and evaluated in melanoma and NMSC patients, and was used in two publications in this review.^29^, ^33^ This 10‐item measure includes questions relating to recurrence, appearance, social and emotional impacts, communication with HCPs and sun behaviour. The single scale measure shows promising validity, but requires further evaluation.

The Basal and Squamous Cell Carcinoma Quality of Life (BaSQoL) questionnaire was recently validated^54^ ^,^ ^55^ following rigorous development and refinement using IRT analysis. The BaSQoL has five subscales: worries, appearance, behaviour, diagnosis and treatment, and other people. Though this measure shows promise it has yet to be used in research and postdates any of the reviews.

Generic HRQoL measures such as the EORTC QLQC‐30 and EQ‐5D have been used in the NMSC group^56^, ^57^ but again may lack sensitivity. While it may prove useful for pharmacoeconomic analyses, the EQ‐5D‐5L alone is unlikely to demonstrate the sensitivity for meaningful exploration of specific QoL issues in this group. Finally, with the introduction of immunotherapy for this population, future studies should consider treatment as well as disease specific measures, such as the FACT‐ICM subscale (Functional Assessment of Cancer Therapy–Immune Checkpoint Modulator subscale).^58^

Key to better understanding QoL issues for this group will be selecting the best assessment tools. However, based on the lack of validation in the advanced or metastatic setting, there is no clear gold standard measure to use. One way to establish this, or to inform future development, is to conduct rigorous qualitative research with the target population. This would help elucidate the needs of this underserved group; what QoL impacts are important to them and what aspects of life are most affected. This may be even more pertinent with the introduction of novel treatments whose impact on QoL is not yet fully scrutinized.

### 5.2 Limitations of this review

This review has some limitations. It is possible that the choice and combination of search terms and our eligibility criteria limited the number of included articles. Our choice to exclude those which focussed solely on measure development or validation resulted in the exclusion of 6 publications. However, these articles would have also been excluded on the basis of study population. It is perhaps questionable whether the two publications by Wali and colleagues^29^, ^33^ fit the inclusion criteria, as their purpose was to assess the feasibility of using the SQOLIT, rather than having QoL as the primary outcome. These publications were retained because they were not development or validation papers and they reported QoL scores.

We have argued that this patient population will have different needs and QoL outcomes to cSCC patients with a favourable prognosis. To take this argument to its logical conclusion, the needs and QoL outcomes of high‐risk patients likely differ from those with advanced disease and warrant separate investigation. For the purpose of this review, we grouped them together due to the few published studies available. The high‐risk cohort have previously been difficult to define without strong standardized guidance.^6^, ^7^ This lack of clarity may have resulted in the small amount of publications seen here. BAD produced a comprehensive classification of low, high and very high‐risk patients at the end of 2020.^7^ This may aid clearer distinction of patient groups and their respective QoL outcomes in future research.

## 6 CONCLUSION

The published body of evidence is small and disparate. It is not possible to tell a coherent story of QoL in advanced/high‐risk cSCC because what little data have been published varies so significantly in terms of the study population, study design and quality. While the identified studies suggested relatively robust QoL, the choice of assessment tools may not be optimal and extrapolation to the cSCC population is not yet warranted. There is a clear need for well designed, longitudinal studies for this patient group.

## CONFLICT OF INTERESTS

No conflict of interests have been declared.

## Supporting information

Supporting Information 1

Supporting Information 2

## Data Availability

Data sharing is not applicable to this article as no new data were created or analysed in this study.
