# Supplementary material for: A structured review of quality of life in advanced and high‐risk cutaneous squamous cell carcinoma shows the need for more studies and better measures
Source: Skin Health Dis. 2021 May 7;1(3):e39. doi: 10.1002/ski2.39 (PMC9060136; doi:10.1002/ski2.39)
Supplement: Supplementary file 1 — Supporting Information 1 [file SKI2-1-e39-s002.docx]

**Supplementary information 1: Search strategy as run in medline**

1. (Quality of life or quality-of-life).ti,ab.
2. (wellbeing or well-being).ti,ab.
3. psychol*.ti,ab.
4. experience.ti,ab.
5. impact.ti,ab.
6. or/1-5
7. non-melanoma.ti,ab.
8. Squamous cell carcinoma.ti,ab.
9. or/7-8
10. skin.ti,ab.
11. cutaneous.ti,ab.
12. or/10-11
13. advanced.ti,ab.
14. metastatic.ti,ab.
15. (high risk or high-risk).ti,ab.
16. unresectable.ti,ab.
17. nonresectable.ti,ab.
18. or/13-17
19. 6 and 9 and 12 and 18
20. Limit 19 to english language
