# Supplementary material for: A structured review of quality of life in advanced and high‐risk cutaneous squamous cell carcinoma shows the need for more studies and better measures
Source: Skin Health Dis. 2021 May 7;1(3):e39. doi: 10.1002/ski2.39 (PMC9060136; doi:10.1002/ski2.39)
Supplement: Supplementary file 2 — Supporting Information 2 [file SKI2-1-e39-s001.docx]

| **Abbreviation** | **Full name** | **Measure background** |
| --- | --- | --- |
| BaSQoL ^(53, 54)^ | Basal and Squamous Cell Carcinoma Quality of Life | Five subscales of worries, appearance, behaviour, diagnosis and treatment, other people. This is a newer measure not yet used in research at the date of this review. |
| DLQI ^(49, 50)^ | Dermatology Life Quality Index | Ten items focusing on daily activities and relationships. Not developed specifically for skin cancer. |
| EORTC QLQ-C30 ^(35, 55)^ | European Organization for Research and Treatment of Cancer Quality of Life questionnaire | Measures QoL in cancer patients via 5 functional scales, a global QoL scale, 3 symptom scales and 6 single items. |
| EQ-5D ^(34)^ | EuroQoL 5-Dimension | Measures generic health related quality of life across - mobility, self-care, usual activities, pain/discomfort and anxiety/depression. |
| FACE-Q skin cancer module ^(51)^ | N/A | Five scales covering appearance satisfaction, quality of life and the patient experience. Emphasis on surgery for early cancer. |
| FACT-G ^(36)^ | Functional Assessment of Cancer Therapy - General | Measures QoL across 4 domains - physical well-being, social and family well-being, emotional wellbeing and functional wellbeing. |
| FACT-H&N ^(37)^ | Functional Assessment of Cancer Therapy – Head and Neck | In addition to the FACT-G, this subscale contains 11 further items relevant to head and neck cancer. |
| FDI ^(41)^ | Facial Disability Index | Ten-item scale of facial motor disorder. This was not developed for skin cancer. |
| POS H/N ^(52)^ | Patient Outcomes of Surgery – Head/Neck | Used to evaluate outcomes for head and neck lesions, both before and after surgery. Not developed for use with advanced patients. |
| SCI ^(8,9)^ | Skin Cancer Index | 15 item measure with 3 subscales of emotional, social and appearance questions. Not included in any study within this review. |
| SCQOLIT ^(38)^ | Skin Cancer Quality of Life Impact Tool | Developed in melanoma and NMSC. Single scale with items relating to recurrence, appearance, social and emotional impacts, communication and sun behaviours. |
| Skindex-16 ^(39, 40)^ | N/A | 16 items across 3 scales of emotions, functioning and symptoms. This was not developed specifically for skin cancer. |
